# Comparative study of internal fixation and external fixation after osteotomy of hallux valgus: A protocol for meta-analysis of comparative studies

**DOI:** 10.1097/MD.0000000000032298

**Published:** 2022-12-23

**Authors:** Wei Guo, Xing Zhou, Wei Dong, Jingfan Yang, Jiankun Chen, Weitong Liu, Hong Yin, Jinlei Li, Rujie Zhuang

**Affiliations:** a Traditional Chinese Medicine Hospital of Shizhu, Chongqing City, China; b Department of Orthopaedics, The First Affiliated Hospital of Zhejiang Chinese Medical University, Hangzhou City, Zhejiang, China; c The First Clinical College, Zhejiang Chinese Medical University, Hangzhou City, Zhejiang, China; d Kunming Municipal Hospital of Traditional Chinese Medicine, Kunming City, Yunnan Province, China; e Kunming University of Science and Technology Hospital, Kunming City, Yunnan Province, China; f Quzhou Traditional Chinese Medicine Hospital, Quzhou City, Zhejiang Province, China.

**Keywords:** external fixation (EF), hallux valgus (HV), internal fixation (IF), protocol

## Abstract

Hallux valgus (HV) is a common clinical deformity of the forefoot, primarily a deformity of the 1st metatarsophalangeal joint in which the bunion is deflected laterally relative to the 1st metatarsal, often in combination with a medial bunion or pain at the head of the 1st metatarsal. For HV bunions that do not respond to conservative treatment, surgical intervention is required, which generally involves osteotomy of the first metatarsal or the first phalanx. However, the choice of fixation method after osteotomy is controversial. Most scholars choose screws or plates for internal fixation (IF) to achieve strong and reliable fixation, while some experts do not perform IF after osteotomy, but reposition the osteotomized end and perform external fixation (EF) with a figure-of-eight bandage between the 1st and 2nd toes, which has been advocated by some scholars because it requires only local anesthesia and has the characteristics of minimally invasive and no additional material for IF, and has achieved good clinical results. Therefore, it is necessary to compare the choice of IF or EF after HV osteotomy to evaluate whether there is a difference between the 2 and to conduct a meta-analysis to provide a reliable basis for clinical guidance.

**Methods:**

We will search articles in 7 electronic databases including Chinese National Knowledge Infrastructure, Wanfang Data, Chinese Scientific Journals Database, Chinese databases SinoMed, PubMed, Embase, and Cochrane Library databases. All the publications, with no time restrictions, will be searched without any restriction of language and status, the time from the establishment of the database to October 2022. We will apply the risk-of-bias tool of the Cochrane Collaboration for randomized controlled trials to assess the methodological quality. Risk-of-Bias Assessment Tool for Non-randomized Studies was used to evaluate the quality of comparative studies. Statistical analysis will be conducted using RevMan 5.4 software.

**Results:**

This systematic review will evaluate the functional outcomes and radiographic results of internal versus EF after HV osteotomy.

**Conclusion:**

The findings of this study will provide evidence to determine whether IF or external fixation is more effective after HV osteotomy.

## 1. Introduction

Hallux valgus (HV) is a condition in which the deformity of the first metatarsophalangeal joint is the main manifestation, and the unsightliness, pain and functional impairment caused by the deformity are the main reasons why patients come to the clinic.^[1]^ The prevalence of HV can be as high as 35%, and approximately 30% of patients undergo surgery for foot pain and discomfort.^[2,3]^ The gold standard for surgical treatment of HV has not yet been fully unified,^[4]^ and the mainstream treatment option is osteotomy, which is mainly performed by various forms of osteotomy of the 1st metatarsal and 1st proximal phalanx or by combined osteotomy of the metatarsal and phalanx to correct the HV deformity. There are various ways of osteotomy and post-osteotomy fixation, but how to achieve the best outcome with minimal trauma has been the focus of HV research.^[5]^

Internal fixation (IF) with hollow screws or plates after HV osteotomy is currently the mainstream approach.^[6,7]^ Most scholars believe that strong IF at the osteotomy end is required after correction of the deformity to avoid postoperative loss of correction angle and to reduce the probability of postoperative recurrence.^[8–13]^ However, some experts believe that strong IF is not always needed after osteotomy, but only external fixation (EF) between the 1st and 2nd toe after correction of the deformity.^[14,15]^ It is believed that the figure-of-eight bandage of EF can be adjusted for tightness, as IF is not needed only for minimally invasive incision, which protects blood flow and avoids soft tissue damage, maintains the stability of the fracture at the osteotomy end, and also allows a moderate amount of micro-movement at the osteotomy end, which maintains the elasticity of the fracture was fixed with good clinical results.^[16–18]^

In this study, we attempted to conduct a meta-analysis of related studies to evaluate and compare the mode of fixation of the osteotomy end after HV osteotomy, and to analyze the radiographic performance, function and complications after IF and EF of the osteotomy end to provide high-level clinical guidelines and evidence-based medical evidence to guide clinical decision-making and application.

## 2. Methods

### 2.1. Study registration

We have prospectively registered this research at the international Prospective Register of Systematic Reviews, registration number: CRD42022374125. We performed this protocol based on the Preferred Reporting Items for Systematic Review and Meta-Analysis Protocols (PRISMA) statement guidelines.^[19]^

### 2.2. Inclusion criteria

#### 2.2.1. Type of participants.

The participants with HV deformity requiring osteotomy surgery will be included regardless of their country, ethnicity, sex and occupation.

#### 2.2.2. Type of interventions.

In the experimental group, all patients received IF after HV osteotomy, including screws, plates, etc. In the control group, all patients received EF after HV osteotomy, such as figure-of-eight bandage EF.

#### 2.2.3. Type of outcome measurements.

##### 2.2.3.1. Primary outcomes.

Clinical and radiological outcomes will be assessed based on the following criteria:

Clinical outcomes measured with

① Pre and postoperative American Orthopaedic Foot and Ankle Society score.
② Pre and postoperative visual analog scale score.

Radiological outcomes measured with

① Pre and postoperative HV angle.
② Pre and postoperative inter-metatarsal angle.

##### 2.2.3.2. Secondary outcomes.

Duration of surgery, cost and complications will be defined as secondary outcomes.

#### 2.2.4. Type of studies.

We will include comparative studies which published in Chinese or English, such as randomized controlled trials, retrospective studies and cohort studies. Review, case reports, experimental studies, expert experience, animal studies and conference abstracts will be excluded.

### 2.3. Search strategy

Chinese National Knowledge Infrastructure, Wanfang, Chinese Scientific Journals Database, Chinese databases SinoMed, PubMed, Embase, and Cochrane Library databases were searched for this study, using the keywords “hallux valgus,” “bunions,” “osteotomy,” ”orthopedic,” “scarf,” “Reverdin-Isham,” “Chevron,” “akin,” “bandage,” “fixation,” and “surgery.” The search strategy in PubMed is shown in Table 1. In addition, the reference lists of previously published systematic reviews of ligament reconstruction for ankle instability were manually examined for further pertinent studies.

**Table 1:** PubMed database search strategy.

Search number	Items
1	“hallux valgus” [MeSH]
2	hallux valgus [Title/Abstract]
3	bunions [Title/Abstract]
4	1 OR 2 OR 3
5	osteotomy [Title/Abstract]
6	orthopedic [Title/Abstract]
7	scarf [Title/Abstract]
8	Reverdin-Isham [Title/Abstract]
9	Chevron [Title/Abstract]
10	akin [Title/Abstract]
11	bandage [Title/Abstract]
12	fixation [Title/Abstract]
13	surgery [Title/Abstract]
14	5 OR 6 OR 7 OR 8 OR 9 OR 10 OR 11 OR 12 OR 13
15	4 AND 14

MeSH = Medical Subject Headings.

### 2.4. Study selection

**Figure 1.**
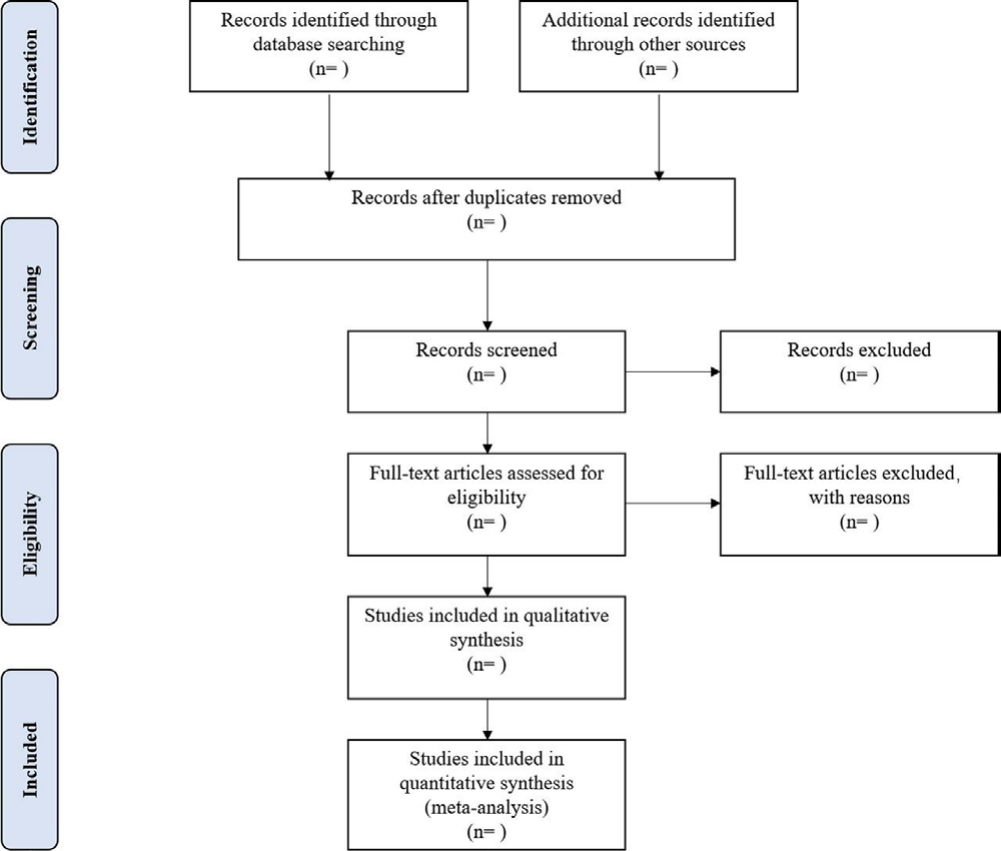
Flowchart of literature selection.

Different researchers will separately screen the titles and abstracts of records acquired potential eligibility which comes from the electronic databases. The obtained literature is managed by Notoexpress, irrelevant and duplicate articles are excluded by reading the title and abstract, full text screening and data extraction will be conducted afterward independently, and finally selected according to the inclusion criteria. Any disagreement will be resolved by discussion with the third author until consensus is reached or by consulting a third author. PRISMA flowchart will be used to show the selection procedure (Fig. 1).

### 2.5. Data extraction and management

The following data were extracted: lead author, publication year, country of origin, study design, sample size, age, mode of fixation, outcome measures, and complications. Any differences of opinion will be resolved through group discussion or consultation with a third reviewer. When relevant data is not reported, we will contact the author via email or other means to obtain missing data. PRISMA flow diagram will be filled out after the screening study is completed to provide specific information.

### 2.6. Risk of bias assessment

Two independent investigators evaluated the quality of the included studies. The Cochrane Collaboration Risk of Bias Tool was used to evaluate the quality of the randomized controlled trials. The methodological quality of the non-randomized studies was assessed using the Risk-of-Bias Assessment Tool for Non-randomized Studies. The level of evidence was assessed according to the Oxford Centre for Evidence-based Medicine Levels of Evidence.

### 2.7. Data synthesis

Statistical analysis will be conducted using RevMan 5.4 software (Cochrane Collaboration) https://www.cochranelibrary.com/search. The mean difference will be used as the effect analysis statistic for continuous variables, while the risk ratio will be used as the effect analysis statistic for categorical variables. We will also calculate 95% confidence interval for each statistic, and summarize statistical heterogeneity among summary data using the *I*^2^ statistic. Cases with *I*^2^ ≤ 50% will not be considered to have significant heterogeneity, thus a fixed-effects model will be applied for meta-analysis. In cases where there is statistical heterogeneity among studies, we will further analyze the source of heterogeneity. A random-effects model will be used to pool the data, after excluding the obvious source of clinical heterogeneity, and in cases where obvious clinical heterogeneity exists, the researchers will perform subgroup, sensitivity or only descriptive analyses. Study-specific and pooled estimates will be graphically presented using forest plots, and *P* < .05 considered statistically significant.

### 2.8. Subgroup analysis

Subgroup analysis according to the age, type of studies and gender will be performed to find the source of heterogeneity when significant clinical heterogeneity is observed.

### 2.9. Sensitivity analysis

Sources of heterogeneity were assessed by sensitivity analysis, by excluding studies of low quality or small sample size, if the heterogeneity did not change significantly, the results were robust, otherwise, the excluded studies may have been sources of heterogeneity.

### 2.10. Publication bias

In this study, fewer than 10 included studies were evaluated for publication bias using funnel plot, otherwise Egger regression test would be used.^[20,21]^

### 2.11. Ethics and dissemination

No ethical approval is required because the study will be a review of literature and will not obtain data from a single patient. We will publish our findings through a peer-reviewed journal.

## 3. Discussion

The purpose of this study was to comparatively assess the final functional outcome and complications of 2 fixation modalities after HV osteotomy. As medical technology advances, patients seek the best outcomes and least pain, and the surgeon seeks the best technology for the patient, this study hopes to provide useful, high-grade evidence-based medical evidence for patients and clinicians to inform better decisions.

## Author contributions

**Conceptualization:** Xing Zhou, Wei Dong, Rujie Zhuang.

**Data curation:** Wei Guo.

**Formal analysis:** Jiankun Chen.

**Funding acquisition:** Jingfan Yang, Jinlei Li.

**Investigation:** Wei Dong, Jingfan Yang.

**Methodology:** Xing Zhou.

**Project administration:** Jinlei Li.

**Software:** Xing Zhou.

**Supervision:** Jiankun Chen, Rujie Zhuang.

**Validation:** Wei Guo, Hong Yin.

**Visualization:** Liu Weitong.

**Writing – original draft:** Wei Guo.

**Writing – review & editing:** Rujie Zhuang.
